# Material Characterization of PCL:PLLA Electrospun Fibers Following Six Months Degradation In Vitro

**DOI:** 10.3390/polym12030700

**Published:** 2020-03-21

**Authors:** Alyah H. Shamsah, Sarah H. Cartmell, Stephen M. Richardson, Lucy A. Bosworth

**Affiliations:** 1 Department of Materials, Faculty of Science and Engineering, The University of Manchester, Oxford Road, Manchester M13 9PL, UK; alyahsaleem1980@gmail.com (A.H.S.); sarah.cartmell@manchester.ac.uk (S.H.C.); 2 Division of Cell Matrix Biology and Regenerative Medicine, School of Biological Sciences, Faculty of Biology, Medicine and Health, The University of Manchester, Manchester Academic Health Science Centre, Oxford Road, Manchester M13 9PL, UK; s.richardson@manchester.ac.uk; 3 Department of Eye and Vision Science, Institute of Ageing and Chronic Disease, Faculty of Life Science, University of Liverpool, Liverpool L7 8TX, UK

**Keywords:** electrospinning, annulus fibrosus, polycaprolactone, poly(L-lactic) acid, polymer blend, degradation

## Abstract

The annulus fibrosus—one of the two tissues comprising the intervertebral disc—is susceptible to injury and disease, leading to chronic pain and rupture. A synthetic, biodegradable material could provide a suitable scaffold that alleviates this pain and supports repair through tissue regeneration. The transfer of properties, particularly biomechanical, from scaffold to new tissue is essential and should occur at the same rate to prevent graft failure post-implantation. This study outlines the effect of hydrolytic degradation on the material properties of a novel blend of polycaprolactone and poly(lactic acid) electrospun nanofibers (50:50) over a six-month period following storage in phosphate buffered saline solution at 37 °C. As expected, the molecular weight distribution for this blend decreased over the 180-day period. This was in line with significant changes to fiber morphology, which appeared swollen and merged following observation using Scanning Electron Microscopy. Similarly, hydrolysis resulted in considerable remodeling of the scaffolds’ polymer chains as demonstrated by sharp increases in percentage crystallinity and tensile properties becoming stiffer, stronger and more brittle over time. These mechanical data remained within the range reported for human annulus fibrosus tissue and their long-term efficacy further supports this novel blend as a potential scaffold to support tissue regeneration.

## 1. Introduction

Clinical use of biomaterials in the body is to repair or restore damaged or diseased tissue [1]. The use of polymeric biomaterials in tissue engineering and as medical devices has been widely extended in recent decades. Biodegradation is a term used to describe the process of breakdown of a material by nature; however, in the case of tissue engineering and regenerative medicine, biodegradation is focused on the biological process inside the body that causes a gradual breakdown of the materials [2]. Biodegradable polymers are commonly used to fabricate implantable biomaterial scaffolds because they are intended to provide a temporary structure that initially supports cells and subsequent tissue regeneration to a point where the newly formed tissue is then mechanically robust enough to withstand applied physiological forces without the aid of a foreign material [3,4,5,6,7,8]. Ideally, the rate of polymer degradation should coincide with that of new tissue, to a point where the tissue is mature enough to resist mechanical loads without causing it to rupture. 

Polyesters, such as polycaprolactone (PCL) and poly(L-lactic) acid PLLA (and their blends) are biodegradable polymers that are regulatory-approved for use in humans and have been widely applied in tissue engineering and as medical devices due to their degradation properties [3,9,10]. They are semicrystaline hydrophobic polyesters with slow degradation profiles in vivo, that range from several months up to five years [11,12]. The degradation of polyesters is caused by cleavage of the ester group by hydrolysis. Degradation of PCL, PLLA and their blends by hydrolysis in vivo occurs due to the breakdown of the ester group in polymeric chains as a result of penetration of biological fluids (or water molecules), leading to polymer scission that cuts long polymer chains into shorter water-soluble fragments, which are then free to hydrogen bond with water molecules and can be subsequently metabolized by the body [4]. Hydrolysis occurs initially within the amorphous regions of the polymer, which are more permeable to water penetration triggering random scission of the ester bonds. The release of acidic breakdown molecules (due to breakdown) causes auto-catalysis to occur, which consequently accelerates the rate of degradation and also initiates attack upon the polymer’s crystalline regions, where the highly ordered chain structures are subsequently broken down [3,4,5,6,7]. 

Our previous studies focused on the development of aligned electrospun fibres made from a novel blend of PCL and PLLA (50:50) that can be used for tissues, such as the annulus fibrosus (AF), located within the intervertebral disc [13,14]. A 3D-ringed tissue with a purposefully oriented fibrous architecture, the AF is susceptible to ageing and disease, which can lead to its herniation and cause chronic lower back pain. In order to regenerate AF tissue and restore its function, a scaffold is required to provide a temporary structure that facilitates cell attachment and proliferation and subsequent matrix deposition until this engineered tissue has reached maturation. We have demonstrated these scaffolds yield tensile properties within the range of human AF and support cell viability and collagen matrix deposition when cultured within 3D ringed structures. However, degradation is an important characteristic for AF tissue engineering, where a strong and stiff biomaterial with a long-term degradation rate (in excess of six months) would be considered suitable to support tissue remodeling and complete replacement with mature tissue [15,16].

Consequently, a six-month degradation study of electrospun PCL:PLLA 50:50 scaffolds was undertaken in vitro to determine the potential impact on material properties and how this could affect its suitability for tissue engineering the AF.

## 2. Materials and Methods

### 2.1. Electrospinning and Scaffold Preparation

Polycaprolactone (PCL; Purac Biomaterials, Amsterdam, The Netherlands) and poly-L-lactic acid (PLLA; Purac Biomaterials, Amsterdam, The Netherlands) (inherent viscosity = 1.3 dL/g and 3.2 dL/g, respectively) were dissolved in 1,1,1,3,3,3-hexafluoro-2-propanol (HFIP; Sigma-Aldrich, United Kingdom) at room temperature to create a 10% w/v of PCL solution and 5% w/v of PLLA solution. From those solutions, a 50:50 PCL:PLLA blended solution was prepared and subsequently electrospun to fabricate aligned nanofibrous scaffolds as previously described by Shamsah et al. [13] Briefly, fibrous scaffolds were collected using a single needle targeted towards a rotating mandrel (width = 120 mm, diameter = 30 mm) using a custom-built electrospinner with the following parameters: continuous flow rate (Cole-Parmer, St. Neots, UK): 1 mL/h, tip-to-collector distance: 20 cm, applied DC voltage (Glassman High Voltage, Reading, UK): 20 kV, and mandrel speed: 600 rpm. Scaffolds were prepared from 100% PCL and 100% PLLA solutions using the same parameters, and solution concentrations were used as control groups. Within a class II biological safety cabinet (Cole-Parmer), all scaffolds were disinfected by fully submerging in 70% v/v ethanol in distilled water and washed twice in phosphate buffered saline (PBS; Invitrogen) before set-up for the degradation study. 

For the degradation study, samples (n = 5) for material characterization were cut from the collected fiber sheet into 10 × 10 mm^2^ squares and subsequently placed in sterile 3 mL microfuge tubes (ThermoFisher, Paisley, United Kingdom) containing 2 mL sterile PBS. For mechanical testing, samples (n = 8) were cut to 23 × 5 mm^2^ (direction of fiber alignment was running parallel to the longer cut length) and suspended in sterile 15 mL centrifuge tubes (ThermoFisher, Paisley, United Kingdom) containing 3 mL sterile PBS Solution. All tubes were covered with a parafilm layer (Sigma-Aldrich, Gillingham, United Kingdom) and held upright within plastic tube racks of appropriate size. Racks were subsequently housed within an automated rotary shaker (100 rpm) chamber with temperature controlled at 37 °C (Cole-Parmer, St. Neots, UK). The degradation study was performed over a 6-month period, with time points at 0, 30, 90 and 180 days. At each time point, samples were removed using forceps and left to air-dry at room temperature for 12 h before testing. 

### 2.2. Material Characterizations

#### 2.2.1. Scanning Electron Microscopy (SEM)

Fiber morphology was evaluated using SEM (Hitachi S-3000N, Berkshire, United Kingdom). Electrospun fiber scaffolds were individually mounted on SEM stubs with carbon tabs and coated with platinum (10 nm layer thickness). Fiber diameter (n = 50) was measured using high magnification images (×6000, EDAX-AMETEK Material Analysis Division, New-Jersey, USA) and Image J software (1.48v, LI-COR Technology, Nebraska, USA). As per [17], fibers with defined edges were included in the analysis and the measurement of merged fibers excluded, as these were deemed too subjective.

#### 2.2.2. Differential Scanning Calorimetry (DSC)

At all time points, scaffolds were tested using DSC (Q20, TA Instruments, Newcastle, United States) under nitrogen gas flow (flow rate = 50 mL/min) and their percentage crystallinity analyzed. For each time point the first heat cycle was used to quantify the Enthalpy of Fusion, from which the percentage crystallinity was calculated. Scaffolds (n = 5) were heated to 250 °C at a rate of 10 °C/min, Enthalpies of Fusion were compared to those for 100% crystalline PCL (139.5 J/g) [18] and 100% crystalline PLLA (93 J/g) [17].

#### 2.2.3. Gel Permeation Chromatography (GPC)

Molecular weight changes over the study period investigated were obtained using GPC (Applied Chromatography Systems, Ltd., Cheshire, United Kingdom), which had been previously calibrated with polystyrene standards in tetrahydrofuran (THF; Fisher Scientific, Paisley, United Kingdom) at known molecular weights (ranging from 600 to 7.7 × 10^6^ g·mol^−1^) and a Phenogel 5 μm column with pore sizes 500 Å, 5 × 10^4^ Å and 5 × 10^6^ Å. Distilled THF with flow rate 1 mL·min^−1^ was used as the mobile phase. At each time point, specimens (n = 5) were individually placed in plastic coated glass bottles (2 mL, Fisher Scientific, Paisley, United Kingdom) and dissolved in THF at room temperature to give a final concentration of 0.2% w/v. Samples were filtered through a 0.45 μm syringe filter and then 100 μL was injected into the GPC column using a 1 mL glass syringe (Adelphi) with a stainless-steel needle (injections were performed in triplicate per sample). Quantitative data were obtained and analyzed using PSS-Win GPC software (PSS-Win, version 2011). 

#### 2.2.4. Mechanical Testing

The tensile properties of the scaffolds were analyzed at each time point (n = 8) as outlined previously by Shamsah et al. [13]. When dry, samples were mounted onto paper windows (25 × 8 mm^2^) using sticky tape with the long edge of the scaffold parallel to the longer length of the window. The shorter edges of the paper windows were subsequently gripped within tensile grips of an Instron (Model 1122), providing a 20 mm gauge length. The paper sides were cut and uniaxial tension was applied using a 10 N load cell and 0.1% strain rate. Stress-strain curves were obtained for each sample and, using the sample’s cross-sectional area, the Young’s modulus, ultimate tensile strength, and maximum percentage strain were calculated. The modulus was determined by comparing two stress-strain points within the linear region of the curve; ultimate tensile stress and maximum strain were taken from the highest points reached on the curve.

### 2.3. Statistical Analysis

The data were statistically analyzed using Graphpad Prism software (v8.1.1) and checked for normality. Parametric data were presented as mean ± standard deviation and non-parametric as median with interquartile range. Statistical analyses were performed using two-way ANOVA followed by Dunnett’s multiple comparisons test (p < 0.05 was considered significant).

## 3. Results

### 3.1. Fiber Morphology and Diameter 

#### 3.1.1. Fiber Morphology 

**Figure 1:**
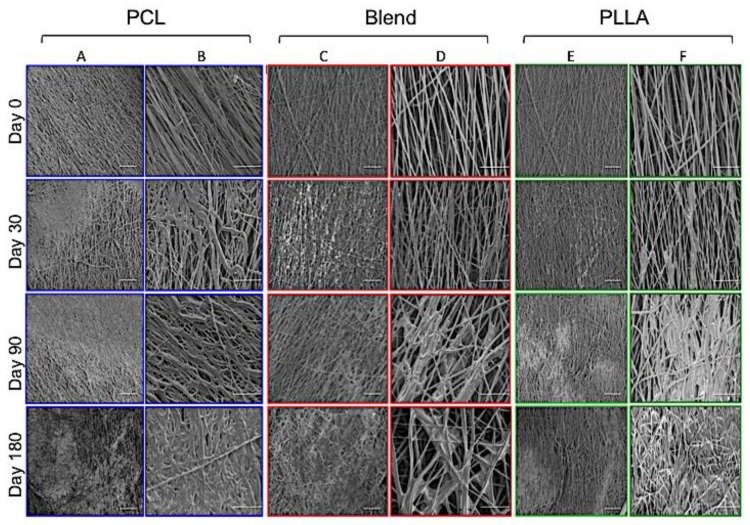
Scanning electron microscopy micrographs of polycaprolactone (PCL) (**A** and **B**), 50:50 PCL:PLLA (poly(L-lactic) acid) blend (**C** and **D**) and PLLA (**E** and **F**) electrospun fibers at time zero and following incubation in phosphate buffered saline solution at 37 °C for 30, 90 and 180 days. Columns A, C and E represent low magnification images (×500) (scale bar = 100 μm); columns B, D and F are high magnifications images (×1500) (scale bar = 20 μm).

Changes in fiber morphology for all scaffold types (PCL, blend and PLLA) under hydrolytic degradation conditions using PBS solution at 37 °C were evaluated at day 30, 90 and 180 using SEM (Figure 1). After 30 days in PBS, all samples retained their normal electrospun fibrous shape with the majority of fibers presenting smooth surfaces; however, some fibers had a slightly roughened appearance at random points along their surface. This was apparent for the PLLA and blended scaffolds, but not for the PCL, which remained smooth despite regions of merged fibers being evident. After 90 days incubation for PCL and PLLA scaffolds, several areas of the scaffolds exhibited notably dense regions, where the fibers appeared to have coalesced as though they had partially melted. These regions were barely observed for the blended scaffold at this time. However, after 180 days, all samples had developed these merged fiber regions and were spread over a larger area, which was particularly noticeable in the PCL scaffolds compared to PLLA and the blend. 

#### 3.1.2. Fiber Diameter 

**Figure 2:**
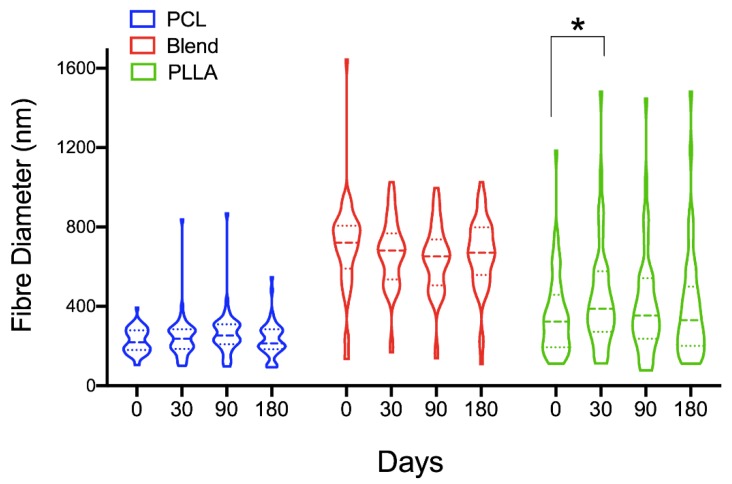
Changes in fiber diameter with time for 50:50 PCL:PLLA blend, PCL and PLLA scaffolds. Statistical comparison performed with two-way ANOVA and Dunnett’s multiple comparisons test (* p < 0.05; n = 50).

Fiber diameters for both degraded and time zero scaffolds were measured and compared (Figure 2). Overall, the majority of fiber diameters for all electrospun scaffolds did not change significantly and remained within the submicron range (<1 μm) after the period of degradation testing was complete. Fibers that had coalesced were excluded from these measurements, as the ability to observe distinct fiber edges was too subjective.

As initially expected, PCL fibers were the finest of the three groups and the blended fibers the largest. There were a visible number of both large and thin diameter fibers present within all of the sample groups over the tested period. PCL fiber diameters were within a relatively tight range at time zero (IQR = 394–105; median = 219 nm) and remained within this range after 30 days incubation. After 90 days however, a 15.5% increase in fiber diameter was observed for PCL, though this was not significant, and after 180 days, the median fiber diameter was 214 nm (IQR = 547–93). For PLLA fibers, there was a significant increase (20.1%) in the median diameter after 30 days incubation compared to time zero (median = 323 nm, IQR = 1185–111 nm). After day 30, a slight decrease occurred at day 90 (9.0%), which then remained relatively constant up to 180 days, reaching a final median value of 330 nm. Blended fibers demonstrated an opposite trend to its individual components, with a slight decrease after 30 days degradation compared to time zero (median = 720 nm; IQR = 1645–135 nm). At day 90, a 4.4% decrease had occurred followed by a slight increase at 180 days, with a median of 671 nm. 

### 3.2. Differential Scanning Calorimetry (DSC)

**Figure 3:**
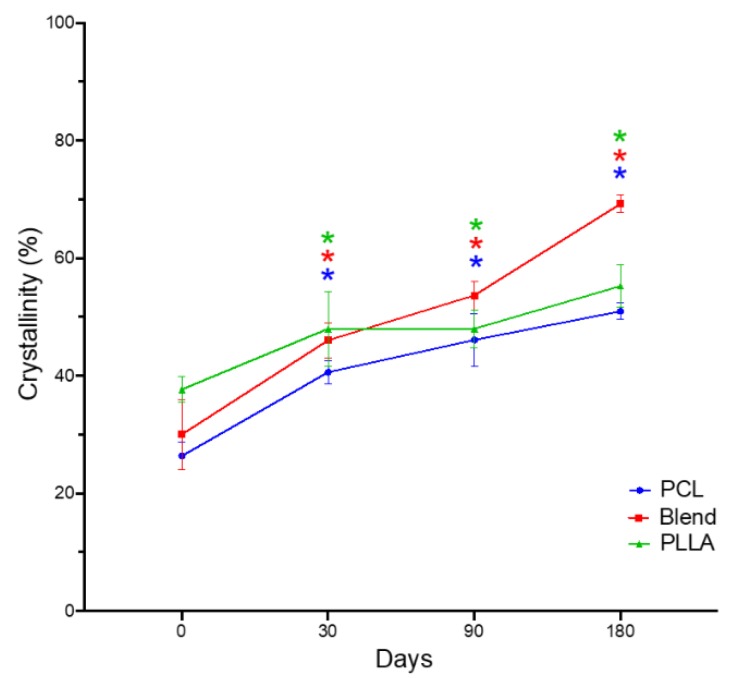
Percentage crystallinities determined from differential scanning calorimetry for PCL, 50:50 PCL:PLLA blend and PLLA electrospun fibers following in vitro degradation in phosphate buffered solution at 37 °C for 180 days. Statistical comparison performed with two-way ANOVA with Dunnett’s multiple comparisons test (* p < 0.05; n = 5). Stars (*) represent statistical significance compared to non-degraded scaffold (time zero).

Percentage crystallinity of all degraded electrospun groups were determined following analysis of each scaffold’s enthalpy of fusion with comparison to known values for pure polymers (Figure 3). Before degradation (at day 0), the percentage crystallinity for PCL (26.4 ± 2.3%) was slightly lower than both the blend (30.0 ± 6.0%) and PLLA (37.7 ± 2.2%) scaffolds. Blended fibers demonstrated a similar trend to their individual components, with a significant increase in percentage crystallinity observed over time, as follows for PCL, blend, and PLLA: after 30 (40.6 ± 2.0%, 46.0 ± 3.0%, 48.0 ± 6.4%), 90 (46.1 ± 4.4%, 53.7 ± 2.4%, 48.0 ± 3.2%) and 180 days (51.0 ± 1.4%, 69.3 ± 1.5%, 55.3 ± 3.6%). 

### 3.3. Gel Permeation Chromatography (GPC)

#### 3.3.1. Weight Average Molecular Weight (Mw)

**Figure 4:**
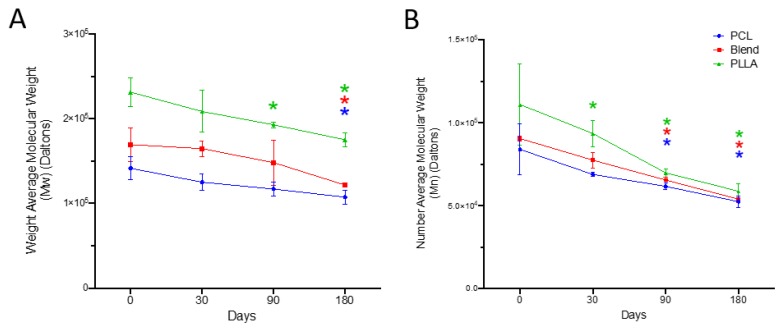
Molecular weight changes for PCL, 50:50 PCL:PLLA blend and PLLA electrospun fibres following storage in phosphate buffered saline at 37 °C for 30, 90 and 180 days and measured using gel permeation chromatography in terms of (**A**) weight average molecular weight (Mw) and (**B**) number average molecular number (Mn). Statistical comparison performed with two-way ANOVA and Dunnett’s multiple comparisons test (* p < 0.05; n = 5). Stars (*) represent statistical significance compared to non-degraded scaffold (time zero).

Molecular mass distributions for all sample groups were measured and compared (Figure 4A). The results revealed a gradual, yet significant, decrease in the weight average molecular weight (Mw) over time for all sample groups (PCL, blend and PLLA) with percentage decreases after 30 days (11.6%, 2.85%, 9.85%, respectively), 90 (17.2%, 12.5%, 16.6%) and 180 days (24.0%, 28.1%, 24.2%), compared to time zero. Greatest loss in Mw was observed for the blended scaffold, with a final Mw of 121.8 ± 2.6 kDa. 

#### 3.3.2. Number Average Molecular Weight (Mn)

Assessment of the number average molecular weight (Mn) for all sample groups (PCL, blends, and PLLA) followed a similar trend to the Mw, with mean values steadily decreasing after 30 (3.2%, 14.3%, 15.6%, respectively), 90 (26.5%, 27.6%, 37.0%) and 180 days (37.5%, 40.4%, 47.0%), compared to time zero (Figure 4B). Greatest loss in Mn was observed for PLLA scaffolds, with a final Mn of 83.3 ± 2.3 kDa. 

### 3.4. Mechanical Properties

#### 3.4.1. Young’s Modulus

**Figure 5:**
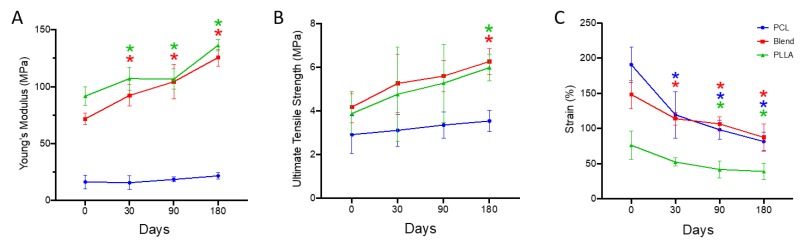
Mechanical properties of PCL, 50:50 PCL:PLLA blend and PLLA electrospun fibers following hydrolytic degradation in phosphate buffered saline solution at 37 °C for 180 days: (**A**) Young’s modulus, (**B**) ultimate tensile strength and (**C**) percentage strain. Statistical comparison performed with two-way ANOVA followed by Dunnett’s multiple comparisons test (* p < 0.05; n = 8). Stars (*) represent statistical significance compared to non-degraded scaffold (time zero).

An overall increase in scaffold stiffness was obtained for all degraded samples compared to time zero following storage in PBS, being significantly different for PLLA and the blended scaffolds (Figure 5A). As expected, the PCL scaffolds (time zero) demonstrated the lowest modulus (16.3 ± 6.0 MPa) compared to other test groups, which increased significantly (32.5%) after 180 days, reaching a final stiffness of 21.6 ± 3.0 MPa. For PLLA scaffolds, a significant increase in stiffness was observed overall, being 91.7 ± 8.0 MPa at time zero and rising to 136.4 ± 5.1 MPa (48.7%) at the final time point. Blended scaffolds presented a similar trend, with a sharp increase measured over time, being 75.4% stiffer and with a final mean modulus of 125.6 ± 7.4 MPa.

#### 3.4.2. Ultimate Tensile Strength

The ultimate tensile strength of the tested groups followed a similar trend to the modulus where an overall increase in strength of the PCL, blend, and PLLA samples was achieved after 180 days (Figure 5B). The PCL samples (time zero) demonstrated the lowest strength value (2.9 ± 0.8 MPa), followed by the PLLA scaffold and then the blended scaffold. A gradual increase for PCL degraded samples was observed over time, being 6.8%, 13.7% and 20.6% after 30, 90 and 180 days, respectively. PLLA at time zero was 3.8 ± 0.9 MPa, and after 30 days a 23.6% increase in strength was observed, followed by a 36.8% and 55.2% rise after 90 and 180 days, reaching 5.2 ± 1.7 MPa and 5.9 ± 0.6 MPa, respectively. For the blended scaffold, a similar trend to PLLA was observed with a sharp rise (26.8%) detected after 30 days (4.1 ± 0.7 MPa). The greatest strength was observed for the blended scaffolds with 5.2 ± 0.3 MPa, 5.5 ± 0.7 MPa and 6.2 ± 0.5 MPa reported following storage in PBS for 30, 90 and 180 days, respectively. 

#### 3.4.3. Maximum Strain

All scaffolds stored in PBS for up to six months demonstrated a significant decrease in maximum strain over time (Figure 5C). As expected, the PLLA scaffolds (time zero) demonstrated the lowest strain percentage (76.1 ± 20.3%) compared to other test groups. The most brittle of the three groups, PLLA scaffolds demonstrated the lowest strain overall, with a final strain of 38.9 ± 11.6% reported after storage for 180 days. The greatest strain (190 ± 25.3%) was observed for PCL scaffolds at time zero, which was significantly reduced by 37.3% following 30 days incubation (119.3 ± 33.1%). PCL scaffolds became less ductile over time, with maximum strain at break being 81.4 ± 13.2% (180 days). Blended samples demonstrated strain behavior similar to those of PCL samples. In comparison to time zero, the blended scaffolds demonstrated a reduction from 148.3 ± 20.1% (time zero) reaching 87.3 ± 19.3% after six months of storage.

## 4. Discussion

Engineering AF tissue in vitro using biodegradable materials requires a balance of new tissue formation and material degradation to ensure a smooth transition of mechanical load following implantation in vivo [19]. As demonstrated in our recent publication [13], 50:50 PCL:PLLA blended scaffolds may be good candidates for use in AF tissue regeneration. The aim of this study was to gain an understanding of the in vitro hydrolytic degradation of our novel polymer blend: 50:50 PCL and PLLA during a six-month period. 

SEM was used to observe morphological changes for all scaffold materials studied (Figure 1). After 30 days submerged in PBS and incubated at 37 °C, visual changes on the surface of the blend and PLLA fibers were observed with fibers appearing rougher. This was in contrast to the PCL fibers, which remained smooth at the first time point investigated. This roughness may be caused by erosion of the fibers’ surface, which—due to their thickened diameters (Figure 2)—possess a larger surface area that is then exposed for hydrolytic attack [20]. Continued incubation up to 90 days demonstrates a change in fiber structure for all scaffold groups, with fibers losing their distinct edges, appearing swollen and/or merging with adjacent fibers. This is most apparent at 180 days with large areas of merged fibers being observed for all groups, though this was most pronounced for PCL scaffolds. These changes in fiber morphology for all groups suggests hydrolysis is also occurring in the bulk of the material [17,18,19]. Measurement of fiber diameter became quite a challenge with increasing incubation time, as it was difficult to distinguish between the edges of fibers. However, fibers that revealed distinct edges were selected for analysis. Interestingly, measurement of these fibers demonstrated no significant change in diameter over time. Fiber diameter for the PCL:PLLA blend remained relatively constant over time with a final median diameter measuring 671 nm (IQR 109–1027 nm) at 180 days, which was a 7% reduction compared to time zero. Furthermore, the distribution of fiber diameters was similar over time, being relatively tight and bimodal for PCL and a greater range for PLLA and the scaffold blend.

A significant increase (*p* < 0.05) in percentage crystallinity was obtained for all groups over time when compared to time zero, with an overall increase of 131% (blended scaffold), 93% (PCL) and 47% (PLLA) following 180 days submersion in PBS at 37 °C (Figure 3). The amorphous regions of semicrystalline polymers are known to more susceptible to hydrolysis, and when this occurs the polymer chains are able to re-orientate to a higher order, which creates new crystalline phases [21]. In the PCL:PLLA blend, variation in the chain size and packing of the molecular chains for PCL and PLLA may cause an increase in the crystallization kinetics to occur, which subsequently affects the formation of crystalline regions. This was demonstrated by Navarro-Baena et al. [22] who reported that crystallization of a PCL:PLLA polymer blend first occurs in the component that is least brittle and with small molecular chain size, leading to the creation of small size crystals (i.e., PCL), and which consequently promotes nucleation of the second component (i.e., PLLA) and the formation of large sized crystals [22]. 

Assessment of GPC data (Figure 4) demonstrated the greatest overall loss in weight average molecular weight (Mw) was for the blended scaffold (26%), followed by PLLA (16%) and then PCL (14%). Similarly, loss in number for average molecular weight (Mn) was observed for all groups, with greatest loss being for PLLA scaffolds (37%) followed by blend (30%) and lastly PCL (23%). This result may confirm the findings of crystallinity, where growth of the crystallizing component in the blended material is related to the presence of two different sizes of crystals (PCL and PLLA) with different molecular weight (Mw) and number (Mn). In addition, blended fibers degrade by random chain scission of the ester groups in the PCL:PLLA material bulk via hydrolysis. This preferentially occurs in the blended polymers’ amorphous regions due to loose structural packing within this area of the polymer [23], making ester bonds more exposed to attack from PBS molecules [24]. Ester bonds are subsequently cleaved from the polymer backbone, resulting in reducing molecular weight and shortening the chain lengths [25]. It is worth noting that the PBS solution was not changed over the course of the study. This could therefore lead to a build-up in carboxylic acid by-products present in solution, which may promote autocatalysis and accelerated degradation of these polymer groups [25,26,27]. Thus, the degradation of the blend may occur over a longer period if housed in an environment that incorporates solution flow, as these acidic breakdown products will be continually removed, which may slow autocatalysis.

Characterization of mechanical properties over time revealed continual increases in both modulus and tensile strength for blended and PLLA scaffolds with final percentage increases in stiffness being 75% (blend) and 49% (PLLA) and in strength being 50% (blend) and 54% (PLLA), compared to time zero (Figure 5A,B). PCL scaffolds, which are notably more ductile and weaker than the PLLA and blend [13], demonstrated similar, albeit subtler, increases with stiffness and strength rising by 33% and 21%, respectfully, after 180 days. These increases may be attributed to the raised levels of crystallinity within these samples over time, as polymer chains within the amorphous regions are able to reorganize and form more stable and ordered structures, which thus enables the material to withstand greater loads [26,28]. Regarding scaffold elongation, PLLA—as expected [13]—yielded the lowest strain values, and the blend behaved similarly to the PCL fiber scaffolds. Over the 180 days investigated, percentage strain demonstrated similar decreasing trends across the three groups with time (Figure 5C). This again may be attributed to recrystallization of the amorphous regions resulting in less flexible and extendable materials, but more work is required to confirm this. 

Retention of mechanical properties upon implantation is necessary to ensure the graft can withstand any load it is subjected to. Comparison of these mechanical data to human AF demonstrated the stiffness of the blended scaffold after 180 days in PBS remained within the reported range for single collagen lamella (59–136 MP [29]), being 125 ± 7 MPa. Comparison of strength also demonstrated the degraded blended scaffold (6.3 ± 0.6 MPa) to be within the range of human AF lamella (3.6–10.0 MPa [29]). These findings suggest our novel 50:50 PCL:PLLA blended scaffold could provide the necessary in vivo mechanical support required until sufficient ingrowth from the natural tissue has occurred. However, further long-term studies where AF fibroblasts are cultured on the blended scaffold are required in order to determine the secretion of the extracellular matrix and its maturation into new tissue versus scaffold degradation. It is most likely that this will require testing in vivo in order to perform a long period of investigation that minimizes risk of infection and incorporates the optimal environment in order to truly evaluate the performance of this scaffold over time. 

## 5. Conclusions

A six-month degradation study of our 50:50 PCL:PLLA electrospun fiber scaffold was undertaken in vitro. To simulate in vivo conditions, scaffolds were fully submerged in PBS solution and incubated at 37 °C within an enclosed orbital shaker. Physicochemical properties of the scaffold were determined. Data suggested no initial changes to the fiber surface of the blend, but fiber morphology at 90 and 180 days demonstrated clear differences, with fibers appearing to coalesce. This suggests hydrolysis was occurring in the bulk of the material, whereby PBS had penetrated the fibers causing them to swell. Over time, significant increases in percentage crystallinity were reflected in the tensile properties, where stiffness and strength of the blend similarly increased with time. These findings are in spite of a fall in molecular weight, demonstrating active hydrolysis of the material, but also remodeling of the amorphous regions to more crystalline phases, which is classic behavior for semi-crystalline polyesters. The overall aim of this study was to determine functionality of the blended scaffold, which has been developed to aid regeneration of the AF. Whilst this in vitro study has provided key information regarding the breakdown of this novel electrospun polymer blend over a significant period of time and suggests material properties continue to be sufficient to support AF tissue regeneration, limitations remain, and a complete understanding of material degradation and tissue response can only be truly evaluated within an in vivo model.
